# Prognostic value of remnant cholesterol in patients with coronary heart disease: A systematic review and meta-analysis of cohort studies

**DOI:** 10.3389/fcvm.2022.951523

**Published:** 2023-01-19

**Authors:** Yun Tian, Wenli Wu, Li Qin, Xiuqiong Yu, Lin Cai, Han Wang, Zhen Zhang

**Affiliations:** Department of Cardiology, The Affiliated Hospital of Southwest Jiaotong University, The Third People’s Hospital of Chengdu, Chengdu, Sichuan, China

**Keywords:** remnant cholesterol, coronary heart disease, prognosis, dyslipidemia, meta-analysis

## Abstract

**Background:**

The relationship between abnormal lipid levels and atherosclerotic cardiovascular diseases is well established, but the association between remnant cholesterol (RC) and coronary heart disease (CHD) remains uncertain. The aim of this meta-analysis is to systematically evaluate the prognostic value of RC concentration in patients with CHD.

**Methods:**

PubMed, EMBASE, Cochrane, and Web of Science databases were reviewed to identify relevant observational cohort studies published in English up to December 2021. Random-effects meta-analysis compared the highest and lowest RC concentration. The primary outcome was a composite of major adverse cardiovascular events (MACEs) and all-cause mortality in patients with CHD.

**Results:**

A total of 10 studies recruiting 30,605 patients with CHD were selected to be included in this meta-analysis. Patients with CHD with elevated RC concentration had an increased risk of the composite endpoint events (RR = 1.54, 95% CI: 1.26–1.87) and MACEs (RR = 1.70, 95% CI: 1.54–1.88), but the risk of all-cause mortality was not statistically significant (RR = 1.16, 95% CI: 0.79–1.69, *P* = 0.44). Subgroup analysis showed consistent results.

**Conclusion:**

Our results suggest that elevated concentration RC may independently predict MACEs in patients with CHD. Determination of RC concentration may improve risk stratification of prognosis in patients with CHD. However, more high-quality studies are necessary to confirm this association.

## Introduction

Coronary heart disease (CHD) is the most common type of organ disease caused by atherosclerosis, which is seriously threatening people’s life and health (1, 2). The prevalence of CHD in the world is approximately 4.6–9.2%, and in 2019, the disease caused 9.14 million deaths worldwide (1). Dyslipidemia is one of the important risk factors for CHD. Currently, lowering low-density lipoprotein cholesterol (LDL-C) is one of the main intervention targets in the treatment of CHD. However, previous studies found that even after reducing LDL-C to an appropriate level and controlling other risk factors, there may be significant differences in the prognosis of patients with CHD (3). Therefore, it is important to find more reliable prognostic indicators to evaluate the long-term prognosis of CHD and formulate the best treatment plan.

In recent years, remnant cholesterol (RC) has been reported to have a critical role in atherosclerosis and CHD, which might indicate it may also be a critical component of the residual risk of patients with CHD (3, 4). RC is the cholesterol content of all non-low-density lipoprotein (LDL) and non-high-density lipoprotein (HDL). Compared with LDL-C, RC had a stronger atherogenic ability because it possesses a larger quantity and volume, carries more cholesterol, and does not need oxidative modification (5, 6). Some observational cohort studies have linked high RC concentrations with an increased risk of CHD (7, 8). Furthermore, RC was found to be causally associated with CHD development in previously healthy individuals (9). However, the prognostic value of plasma RC levels in secondary prevention settings is still undefined because previous studies showed inconsistent and controversial results (10–12). Meanwhile, it is yet to be established whether the prognostic value of RC varies among populations, ages, or the classification of CHDs. In particular, there is still a lack of a standardized method for RC measurement, with strikingly different RC concentrations across studies (10, 12, 13), which may also have contributed to the discrepancy in the outcomes (14–16).

Therefore, our study aims to systematically review and compile meta-analyses of the evidence on the relationship between RC concentration and CHD outcome, by identifying the potential confounders and investigating the prognostic value, which may provide a novel perspective for risk assessment and treatment in patients with CHD.

## Materials and methods

Our meta-analysis was performed according to the recommendation of the meta-analysis of Observational Studies in Epidemiology (MOOSE) (17) and the Preferred Reporting Items for Systematic Reviews and Meta-Analyses (PRISMA) statement (18).

### Search strategy

We comprehensively searched four medical databases, including PubMed, EMBASE, Cochrane, and Web of Science, to identify cohort studies assessing the relationship between RC concentration and cardiovascular outcomes published in English from inception up to 25 December 2021. The query syntax was set using Medical Subject Headings (MeSH) and thesaurus search terms, including (“remnant cholesterol” OR “remnant-like particle cholesterol” OR “triglyceride-rich lipoprotein cholesterol”) AND (“coronary heart disease” OR “coronary disease” OR “coronary artery disease”). The detailed search strategy was presented in the Supplementary material. References retrieved from the studies, as well as relevant reports, were also hand-searched to reduce the likelihood of missing any publications.

### Inclusion and exclusion criteria

The inclusion criteria for this study were presented as follows: (1) cohort studies; (2) an inception cohort involving adults with CHD; CHD included stable or unstable angina, and myocardial infarction (MI); (3) the exposure factor was RC concentration; (4) the endpoint was major adverse cardiovascular events (MACEs) and all-cause mortality. MACEs included cardiac death, MI, ischemic stroke, myocardial ischemia, heart failure, unstable angina requiring readmission, and coronary revascularization; and (5) the highest and lowest RC concentration groups of multivariate-adjusted relative risks (RRs), odds ratios (ORs), or hazard ratios (HRs) and their 95% confidence intervals (95% CI) or the above indicators could be calculated with the complete data (19).

Exclusion criteria included (1) case reports, commentary, and conference abstracts; (2) animal, cross-sectional studies, or randomized clinical trials; (3) studies carried out among pregnant women or children; and (4) examined non-relevant outcomes.

YT did the screening of the titles and abstracts of the identified articles, and pertinent articles were independently reviewed in full text by two investigators (YT and WW). Thus, disagreement was resolved through consensus.

### Data collection and quality assessment

Data extraction was in a standardized style. Two investigators (YT and LQ) independently extracted the following data: the first author, publication year, population, study design, type of CHD, sample size, percentage of women, age, exposure assessment method, fasting status, follow-up duration, outcome assessment, categorical or continuous, adjusted risk estimates, and adjustment for variables.

The quality of observational cohort studies was assessed using the Newcastle–Ottawa Scale (NOS) (20), which was ranked as poor (score 1–3), fair (score 4–6), or good (score 7–9) according to the quality of study participant selection, comparability, and outcome. Studies with NOS ≥7 points were considered high quality. Any disagreements were discussed and resolved by a chief investigator (HW), and a consensus was reached in all cases.

### Statistical analysis

Review Manager 5.4 software (The Cochrane Collaboration, Oxford, UK) and Stata 16.0 (Stata Corporation, College Station, TX, USA) were employed for statistical analysis. The *I*^2^ statistics and chi-square Cochran’s *Q*-test were used to assess the heterogeneity across studies. If *P* ≥ 0.05 and *I*^2^ < 50%, suggesting that no significant heterogeneity could be found, a fixed-effect model was also applied. In addition, if *P* < 0.05 and *I*^2^ ≥ 50%, a random-effect model was cautiously applied, and then subgroup analysis was used to explore the source of heterogeneity (21). The elimination of individual studies one by one was also performed for sensitivity analysis in order to explore the heterogeneity and assess the stability of the meta-analysis. A funnel plot combined with Egger’s test was employed to investigate the potential publication bias of the involved studies. Finally, the RR with 95% CI was employed for the effect estimation metric, and HR and OR were converted into RR (22–24). The *p*-value of < 0.05 meant the difference was statistically significant.

## Results

### Literature search results

**FIGURE 1:**
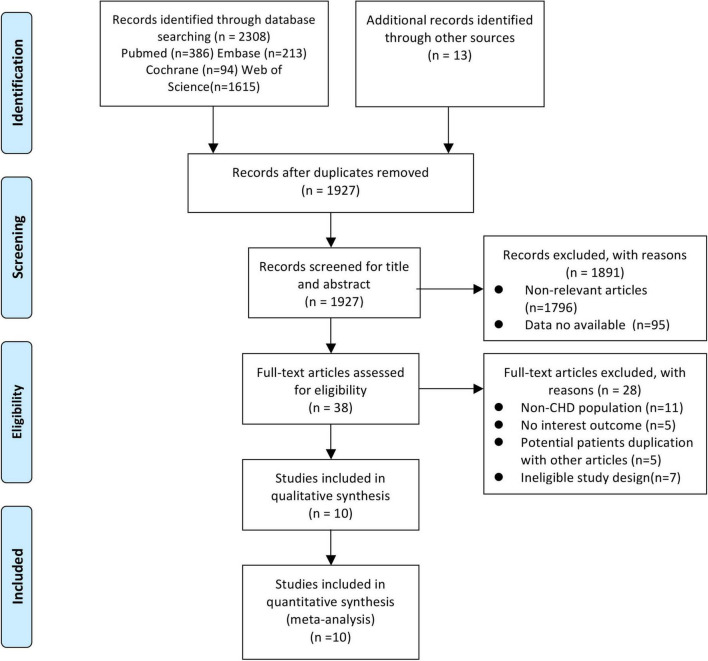
Flow diagram of the study search and selection process. From Moher et al. (65).

A total of 2,308 articles were retrieved, and after 381 duplicates were removed, 1,927 unique records remained. After screening the titles and abstracts of the articles, 38 records were considered for a detailed full-text screening. Of these studies, 28 articles were excluded: studies with a non-CHD population (*n* = 11) (16, 25–34), those with no interest outcome (*n* = 5) (35–39), those with potential patients’ duplication with other articles (*n* = 5) (8, 9, 40–42), and studies with ineligible study design (*n* = 7) (43–49). Finally, 10 articles (4, 10–13, 50–54) covering 12 cohorts enrolling 30,605 subjects met the selection criteria. Figure 1 depicts the literature screening process and results in detail.

### Study characteristics and quality assessment

The main characteristics of the included studies are summarized in Table 1. Of the 10 included studies, three were performed in China (11, 50, 54), three in Japan (4, 13, 51), two in Denmark (52, 53), one in the United States (12), and one collaborative study involving multiple countries (10). The studies were published from 1999 (13) to 2021 (11), of which nine were prospective cohort designs (4, 11–13, 50–54) and one was retrospective cohort design (10). In addition, two studies included two cohorts (50, 52). The sample size ranged from 120 (51) to 6723 (11). The follow-up time ranged from 1.7 (51) to 7.0 (52) years, and participants’ age varied from 57.7 (11) to 68.0 (53) years. The average NOS scores for these studies included were 7.3, demonstrating that the quality of the cohort study was good.

**TABLE 1:** Main characteristics of the included studies.

First author, year	Population	Type of study	Partici-pation	Sample size (number)	% females	Age (year)	RC concen-tration (mg/dl)	Exposure assessment	Fasting status	Follow-up duration (year)	Outcome assessment	OR, RR, or HR (95%CI)	Categorical or continuous	Variables adjusted^1^	NOS score
Cao et al. (50)	Chinese	prospective cohort study	CAD	4355	28.9	58.2 ± 9.7	5.0 (2.7–9.7)	Automated assay	Fasting	5.1	MACEs	HR: 1.53 (1.16–2.02)	Q5 vs. Q1	1, 2, 3, 4, 5, 6, 7, 8, 9, 10, 11, 12	9
Cao et al. (50)	Chinese	prospective cohort study	CAD	4355	28.9	58.2 ± 9.7	9.0 (6.5–12.4)	Immunosepa-ration	Fasting	5.1	MACEs	HR: 1.49 (1.12–2.09)	Q5 vs. Q1	1, 2, 3, 4, 5, 6, 7, 8, 9, 10, 11, 12	9
Elshazly et al. (10)	North and South American, etc.	retrospective cohort study	CAD	5754	28.0	58.1 ± 9.2	23.8 (19.1–30.8)	Calculation	Fasting	2.0	MACEs	HR 1.62 (1.27–2.07)	Q4 vs. Q1	–	6
Fujihara et al. (4)	Japanese	prospective cohort study	CAD	247	9.0	67 (60–74)	3.6 (2.5–5.5)	Immunosepa-ration	Fasting	3.2	MACEs	HR 1.62 (1.26–2.07)	≥ 3.9 mg/dl vs. < 3.9 mg/dl	4, 11, 13, 14, 15	8
Fukushima et al. (51)	Japanese	prospective cohort study	CAD + DM	120	37.5	65.6 ± 8.4	5.8 (3.1–6.2)	Immunosepa-ration	Fasting	1.7	MACEs	OR 2.2 (1.2–6.4)	> 4.7 mg/dl vs. ≤ 4.7 mg/dl	1, 4, 7, 8, 9, 10, 11, 13, 16, 17, 18	6
Jepsen et al. (52)	Danish	prospective cohort study	IHD	5414	30.3	64.4	14.4 (9.0–21.6)	Calculation	non-fasting	7.0	mortality	HR: 1.5 (1.2–2.0)	Q4 vs. Q1	1, 2, 4, 6, 16	8
Jepsen et al. (52)	Danish	prospective cohort study	IHD	5414	30.3	64.4	1.4 (0.7–3.4)	Automated assay	non-fasting	7.0	mortality	HR: 1.2 (1.0–1.5)	Q4 vs. Q1	1, 2, 4, 6, 16	8
Kugiyama et al. (13)	Japanese	prospective cohort study	CAD	135	34.0	65.0 ± 9.7	3.4	Immunosepa-ration	Fasting	2.2	MACEs	OR 6.38 (2.3–17.6)	highest vs. lowest tertile	1, 2, 4, 5, 8, 10, 11, 16, 19, 20	7
Langsted et al. (53)	Danish	prospective cohort study	MI/IS	2973	32.0	68 (61–74)	NP	Calculation	non-fasting	NR	MACEs	HR 1.71 (1.24–2.36)	Q4 vs. Q1	4, 9, 15, 16, 22	7
Liu et al. (11)	Chinese	prospective cohort study	CAD	6723	26.2	57.7 ± 10.8	9.2 ± 5.0	NP	NP	4.9	MACEs	HR 1.79 (1.18–2.71)	Q4 vs. Q1	1, 2, 3, 4, 5, 6, 7, 9, 10, 11, 12, 16, 18, 21, 33	8
Martin et al. (12)	American	prospective cohort study	AMI	2465	32.0	58 ± 12	20 (14–27)	VLDL3-C + IDL-C	NP	2.0	mortality	HR 0.76 (0.64–0.91)	T3 vs. T1	1, 2, 3, 4, 5, 6, 9, 10, 16, 20, 23, 24, 25, 26, 27, 28, 29, 30, 31, 32, 33, 34, 35, 36	7
Zhao et al. (54)	Chinese	prospective cohort study	NSTE-ACS	2419	28.2	60.08 ± 8.97	12.4 ± 7.6	Calculation	Fasting	3.0	MACEs and mortality	MACEs: HR 1.960 (1.558–2.465); mortality: HR 2.207 (0.612–7.959);	highest vs. lowest	–	7

NP, not provided; HR, hazard ratio; OR, odds ratio; RR, risk ratio; CI, confidence intervals; CAD, coronary artery disease; DM, diabetes mellitus; IHD, ischemic heart disease; MI, myocardial infarction; IS, ischemic stroke; AMI, acute myocardial infarction; NSTE-ACS, non-ST segment elevation acute coronary syndrome; MACEs, major adverse cardiovascular events; T, tertile; Q4, quartile; Q5, quintile; NOS, Newcastle–Ottawa Scale.

^1^Adjustments: age (1), sex (2), body mass index (3), smoking (4), diabetes (5), statin use (6), family history of CAD (7), TC (8), LDL-C (9), HDL-C (10), triglyceride (11), hsCRP (12), HbA1c (13), ApoB (14), lipoprotein (a) (15), hypertension (16), three-vessel disease (17), left ventricular ejection fraction (18), stenosis of left main coronary artery (19), number of diseased coronary arteries (20), creatinine (21), lipid-lowering therapy (22), the GRACE 1.0 score (23), site (24), race (25), insurance (26), education (27), alcohol use (28), physical activity (29), kidney disease (30), heart failure (31), prior MI (32), ezetimibe (33), niacin (34), fibrate (35), and fish oil (36).

### Meta-analysis results

**FIGURE 2:**
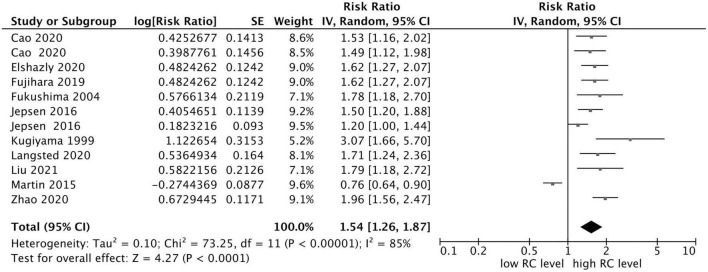
Forest plots showing the pooled RR with 95% CI of composite endpoint events for the highest versus lowest remnant cholesterol concentration.

The results demonstrated that elevated RC concentration was related to an increased risk of composite endpoint events (MACEs and all-cause death) (RR = 1.54, 95% CI: 1.26–1.87, *P* < 0.0001) in a random-effect model (Figure 2). Significant heterogeneity between studies was observed (*I*^2^ = 85%, *P* < 0.0001), and sensitivity analysis indicated that the total combined effect size did not change significantly in each step, demonstrating that the meta-analysis results were relatively stable. However, if the study conducted by Martin et al. (12) was eliminated, the heterogeneity decreased significantly (*I*^2^ = 44%, RR = 1.61, 95% CI: 1.43–1.80).

**FIGURE 3:**
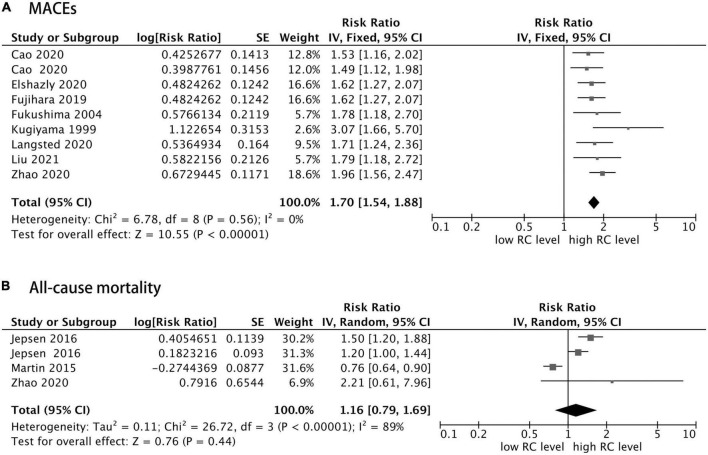
Forest plots showing the pooled RR with 95% CI of major adverse cardiovascular events (MACEs) **(A)** and all-cause mortality **(B)** for the highest versus lowest remnant cholesterol concentration.

In addition, as shown in Figure 3, eight of 10 studies reported the MACEs as an outcome, and the other two studies addressed the all-cause mortality outcome, in which one study reported both MACEs risk and all-cause mortality risk. Furthermore, patients with CHD with elevated RC concentration had an increased risk of MACEs (RR = 1.70, 95% CI: 1.54–1.88, *P* < 0.0001) without significant heterogeneity (*I*^2^ = 0%, *P* = 0.56) in a fixed-effect model. However, the risk of all-cause mortality was not statistically significant (RR = 1.16, 95% CI: 0.79–1.69, *P* = 0.44), with significant heterogeneity (*I*^2^ = 89%, *P* < 0.0001) in a random effect model. In addition, sensitivity analysis showed that after removing the study of Martin et al. (12), the heterogeneity decreased significantly, and the total combined effect size changed as well (*I*^2^ = 32%, RR = 1.34, 95% CI: 1.10–1.63, *P* = 0.003).

### Subgroup analysis

In the subgroup analysis, the association remained constant, suggesting a positive association between RC concentration and CHD risks in studies conducted in the Asian region (RR = 1.72, 95% CI: 1.53–1.95, *P* < 0.0001) for those with the diagnosis of MI (RR = 1.64, 95% CI: 1.46–1.85, *P* < 0.0001) and with older age (≥65 years old) (RR = 1.78, 95% CI: 1.46–2.15, *P* < 0.0001). In addition, this constant association continued in selected studies for those publications with sample size <1,000 cases (RR = 1.88, 95% CI: 1.38–2.55, *P* < 0.0001), those that used the immunoseparation method (RR = 1.72, 95% CI: 1.39–2.12, *P* < 0.0001) and fasting status test (RR = 1.70, 95% CI: 1.51–1.91, *P* < 0.0001) for RC assessment, and studies with a long follow-up time (≥3 years) (RR = 1.54, 95% CI: 1.35–1.76, *P* < 0.0001) (Table 2).

**TABLE 2:** Subgroup analysis on composite endpoint events.

Subgroup	No. of studies	Pooled risk ration	95% confidence interval	*P*-value	Heterogeneity between studies
**Region**					
Asian	6	1.72	1.53–1.95	<0.0001	*I*^2^ = 8.9%, *P* = 0.361
No-Asian	4	1.55	1.25–1.93	<0.0001	*I*^2^ = 63%, *P* = 0.030
**Participation**					
CAD	6	1.64	1.46–1.85	<0.0001	*I*^2^ = 0%, *P* = 0.55
MI	3	1.36	0.70–2.64	0.37	*I*^2^ = 96%, *P* < 0.001
**Sample size**					
≥ 1000	7	1.44	1.15–1.81	0.0020	*I*^2^ = 87%, *P* < 0.001
< 1000	3	1.88	1.38–2.55	<0.0001	*I*^2^ = 44%, *P* = 0.17
**Age**					
≥ 65 years old	4	1.78	1.46–2.15	0.005	*I*^2^ = 88%, *P* < 0.001
< 65 years old	6	1.42	1.11–1.81	0.04	*I*^2^ = 91%, *P* < 0.001
**RC assessment**					
Calculation	4	1.69	1.49–1.91	<0.0001	*I*^2^ = 0%, *P* = 0.42
Immunosepa-ration	4	1.72	1.39–2.12	<0.0001	*I*^2^ = 33%, *P* = 0.21
Automated assay	2	1.32	1.05–1.67	0.0200	*I*^2^ = 52%, *P* = 0.15
**Fasting status**					
Fasting	6	1.70	1.51–1.91	<0.0001	*I*^2^ = 11%, *P* = 0.35
Non-fasting	2	1.41	1.15–1.73	0.0009	*I*^2^ = 55%, *P* = 0.11
**Follow-up duration**					
≥ 3 year	5	1.54	1.35–1.76	<0.0001	*I*^2^ = 50%, *P* = 0.06
< 3 year	4	1.54	0.87–2.74	0.14	*I*^2^ = 93%, *P* < 0.001

### Publication bias test

**FIGURE 4:**
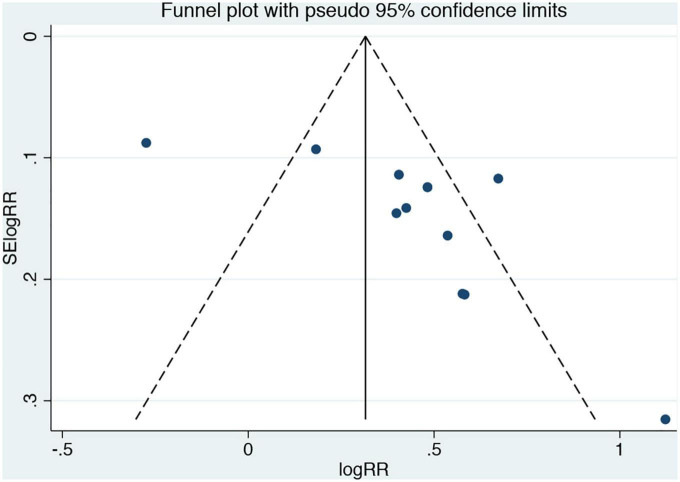
Funnel plots for the analysis of remnant cholesterol concentration and composite endpoint events. Results compare participants in the highest versus lowest remnant cholesterol concentration.

As shown in Figure 4, the funnel plot was asymmetrical, and for further quantitative analysis using Egger’s test, a publication bias was suggested (*P* < 0.05).

## Discussion

In the present study, the relationship between RC concentration and the prognosis of the patients with CHD was evaluated by meta-analysis for the first time. The results illustrated that elevated RC concentration was significantly correlated with an increased risk of the composite endpoint events and MACEs in patients with CHD, but the risk of all-cause mortality was not statistically significant. In addition, the prognostic significance of higher RC concentration on CHD risks was also confirmed in the subgroup analysis. This meta-analysis contributes to the increasing evidence that higher RC concentration may be an independent predictor of poor cardiovascular outcomes in patients with CHD.

Remnant cholesterol, also known as triglyceride-rich lipoprotein cholesterol, is the cholesterol content of all non-LDL and non-HDL. In the fasting state, RC is composed of liver-derived very low-density lipoprotein (VLDL) and intermediate-density lipoprotein (IDL) in the fasting state, as well as intestinal-derived chylomicron remnants (CM) (27). Recently, an increasing number of studies have demonstrated that RC concentration had a relationship to the occurrence and development of atherosclerosis (27, 53). Particularly, when LDL-C was controlled at an appropriate level, RC was assumed to be the main reason for mediating residual risks in the patients with CHD and even a better predictor of risk than LDL-C (52). Unlike LDL-C, RC could easily penetrate the vessel wall and is directly taken up by the scavenging receptors on macrophages without oxidative modification, leading to forming foam cells and promoting atherosclerotic plaque formation (55, 56). In addition, it could also increase the production of reactive oxygen species free radicals, cause endothelial cell dysfunction (57), and induce the expression of pro-inflammatory mediators, as well as the production of cytokines, interleukin, and atherosclerotic adhesion molecules (58). All of the earlier mechanisms can lead to plaque formation and progressive rupture and promote the occurrence of MACEs, which in turn influences the prognosis of the patients.

The previous clinical studies demonstrated similar conclusions between RC concentration and prognosis in the general population but not in patients with CHD. The latest study by Wadstrom et al. (8) revealed that in the Copenhagen General Population Study, during the 15-year follow-up of 106,937 people, elevated RC concentration was relevant to an increased risk of MI up to multivariable-adjusted HR of 4.2, as well as corresponding HRs were 1.8 for ischemic stroke, and 4.8 for peripheral artery disease (PAD). In addition, in the Copenhagen City Heart Study, corresponding HRs were 2.6 for MI, 2.1 for ischemic stroke, and 4.9 for PAD (8). Castañer et al. (27) also reported that in the PREDIMED cohort study of high cardiovascular-risk groups, every 10 mg/dl increase in RC concentration would increase the risk of cardiovascular events by 21%. After multivariate-adjusted analysis, it was concluded that the levels of triglyceride and RC rather than LDL-C were related to the occurrence of MACEs in the population who were overweight or obese and had a high risk of cardiovascular diseases, which were independent of lifestyle and other risk factors. A few other studies have also reached a similar conclusion (32, 33, 59). However, in our study, RC concentration had no effect on the risk of all-cause mortality, possibly due to the available small sample size and high heterogeneity. Furthermore, it seems the results were consistent across the populations and ages in our subgroup analysis. Wang et al. (31) also indicated the importance of preventive efforts across the adult life course. Obviously, these results need further confirmation in more stratified cases.

In addition, genetic evidence has also been found that RC was the risk factor for atherosclerosis. Varbo et al. (9) performed the Mendelian randomization method by detecting the genes of 73,513 people from the Copenhagen study and selected 15 genotypes to observe the incidence of ischemic heart disease (IHD) for each type of gene. The results indicated that for every 1 mmol/l increase of non-fasting RC concentration, the risk of IHD increased by 2.8 times. In another Mendelian randomized trial, Varbo et al. (60) found that elevated RC concentrations in non-fasting status were causally related to inflammation and IHD, whereas increased LDL-C was only related causally to IHD without inflammation. Jørgensen et al. (61) also indicated that genetic variation in ApoA5 related to stepwise increases of the RC concentration and with comparable increases in the risk of MI. Thus, these results illustrated that exposure to elevated RC concentrations caused by genetic abnormalities could bring a greater risk of cardiovascular diseases.

In our study, sensitivity analysis found that the source of heterogeneity might be the research conducted by Martin et al. (12). In this study, the RC evaluation method was significantly different from others, in which the sum of VLDL_3_-C and IDL-C was used to calculate RC and fasting state was unknown. At present, no uniform method to measure RC concentration has been provided, and accurate measurement is still challenging, which might be the main reason for conflict in the findings (14–16). This was mainly because RC was composed of different lipids and lipoproteins. Then, its rapid and continuous catabolism, the size, quantity, density, and composition of lipoprotein residues were highly dynamic, which was difficult to distinguish from its precursors (non-remnant lipoproteins) (47). Currently, the simplest way to estimate RC concentration is through calculation method (62); that is, RC was calculated as total cholesterol (TC) minus LDL-C minus HDL-C [i.e., RC = (TC)–(LDL-C)–(HDL-C)]. Although it was not as accurate as the method to direct the detection of RC, it has been widely applied at present due to its convenience and simplicity (10, 52–54). Apart from the calculation, there were also several direct methods to identify and quantify RC depending on their specific ingredients, such as immunoseparation (4, 13, 50, 51), direct homogenous assays (50, 52), preparative ultrafiltration (63), and nuclear magnetic resonance (64). In our study, subgroup analysis indicated that elevated RC concentration measured by the immunoseparation method had higher cardiovascular risk. Furthermore, whether in a fasting state during the detection also had an impact on the RC measurement. The subgroup analysis of our study indicated that the CHD patients with elevated RC concentrations in the fasting state had a higher risk of poor prognosis. Apparently, no optimal way of accurately quantifying RC measurement currently exists, so there was a lack of uniform RC cut-off levels to define high RC concentration. However, as increasing importance has been attached to RC, a consensus definition of RC with accurate and reproducible quantitative measurement approaches is eagerly required.

## Limitations

This study also had potential limitations. First, the studies included in this study were all published in English, which might have a language bias. Second, the differences in the types of CHD and RC measurement approaches in each study may lead to clinical heterogeneity. Third, the included studies adjusted some confounders, but other unadjusted risk factors may exist. Some traditional CHD factors cannot be extracted adequately from the included studies, which might also lead to bias. Thus, further studies of stratified analysis for the risk factors of CHD outcome are necessary. Fourth, there were insufficient relevant data to compare the prognostic effect between LDL-C and RC from the included studies. Then, it is worth answering this valuable question in future studies. Finally, there was a significant publication bias in our study, suggesting the possible presence of negative results that were not published. Therefore, future studies are needed before a firm conclusion can be drawn concerning the association between RC concentration and CHD outcome.

## Conclusion

This meta-analysis of 10 cohort studies showed that CHD patients with elevated RC concentrations had a higher risk of adverse cardiovascular outcomes. Measurement of RC concentration has the potential to improve risk classification in patients with CHD. However, future larger sample sizes and higher quality studies are still required to confirm the findings.

## Data availability statement

The original contributions presented in this study are included in this article/Supplementary material, further inquiries can be directed to the corresponding authors.

## Author contributions

YT, WW, and LQ performed the literature search, data extraction, and quality assessment. YT performed data analysis and drafted the manuscript. XY and LC critically revised the study. HW and ZZ designed the study, interpreted the data, and revised the manuscript. All authors contributed intellectually to this manuscript and have approved this final version.
