# Supplementary material for: Prognostic value of remnant cholesterol in patients with coronary heart disease: A systematic review and meta-analysis of cohort studies
Source: Front Cardiovasc Med. 2023 Jan 19;9:951523. doi: 10.3389/fcvm.2022.951523 (PMC9892060; doi:10.3389/fcvm.2022.951523)
Supplement: Supplementary file 1 [file Data_Sheet_1.docx]

**Search Strategy**

**Database: Pubmed**

| 1. “remnant cholesterol”[MeSH Terms] |
| --- |
| 1. “remnant-like particle cholesterol”[MeSH Terms] |
| 1. “triglyceride-rich lipoprotein cholesterol”[MeSH Terms] |
| 1. “remnant cholesterol”[Title/Abstract] |
| 1. “remnant-like particle cholesterol”[Title/Abstract] |
| 1. “triglyceride-rich lipoprotein cholesterol”[Title/Abstract] |
| 1. 1 OR 2 OR 3 OR 4 OR 5 OR 6 |
| 1. “coronary artery disease”[MeSH Terms] |
| 1. “coronary heart disease”[MeSH Terms] |
| 1. “coronary disease”[MeSH Terms] |
| 1. “coronary artery disease”[Title/Abstract] |
| 1. “coronary artery diseases”[Title/Abstract] |
| 1. “coronary heart disease”[Title/Abstract] |
| 1. “coronary heart diseases”[Title/Abstract] |
| 1. “coronary disease”[Title/Abstract] |
| 1. “coronary diseases”[Title/Abstract] |
| 1. 8 OR 9 OR 10 OR 11 OR 12 OR 13 OR 14 OR 15 OR 16 |
| 1. 7 AND 17 |

**Database: Embase**

| #1 | 'remnant cholesterol'/exp |
| --- | --- |
| #2 | 'triglyceride-rich lipoprotein cholesterol' |
| #3 | 'remnant-like particle cholesterol'/exp |
| #4 | 'remnant cholesterol':ab,ti |
| #5 | 'triglyceride-rich lipoprotein cholesterol':ab,ti |
| #6 | 'remnant-like particle cholesterol':ab,ti |
| #7 | #1 OR #2 OR #3 OR #4 OR #5 OR #6 |
| #8 | 'coronary artery disease'/exp |
| #9 | 'ischemic heart disease'/exp |
| #10 | 'coronary artery disease':ab,ti |
| #11 | 'coronary artery diseases':ab,ti |
| #12 | 'coronary heart disease':ab,ti |
| #13 | 'coronary heart diseases':ab,ti |
| #14 | 'coronary disease':ab,ti |
| #15 | 'coronary diseases':ab,ti  #8 OR #9 OR #10 OR #11 OR #12 OR #13 OR #14 |
| #16 | #7 AND #15 |

**Database: Cochrane**

1. (remnant cholesterol):ti,ab,kw
2. (remnant cholesterolremnant-like particle cholesterol):ti,ab,kw
3. (triglyceride-rich lipoprotein cholesterol):ti,ab,kw
4. #1 OR #2 OR #3
5. MeSH descriptor: [Coronary Artery Disease] explode all trees
6. MeSH descriptor: [Coronary Disease] explode all trees
7. (coronary artery disease):ti,ab,kw
8. (coronary heart disease):ti,ab,kw
9. (coronary disease):ti,ab,kw
10. #5 OR #6 OR #7 OR #8 OR #9
11. #4 AND #10

**Database: Web of Science**

1. TS=(remnant cholesterol OR remnant-like particle cholesterol OR triglyceride-rich lipoprotein cholesterol)
2. TS=(coronary artery disease OR coronary artery diseases OR coronary heart disease OR coronary heart diseases OR coronary disease OR coronary diseases)
3. #1 AND #2
